# Method for Activity Sleep Harmonization (MASH): a novel method for harmonizing data from two wearable devices to estimate 24-h sleep–wake cycles

**DOI:** 10.1186/s44167-023-00017-5

**Published:** 2023-04-05

**Authors:** Erin E. Dooley, J. F. Winkles, Alicia Colvin, Christopher E. Kline, Sylvia E. Badon, Keith M. Diaz, Carrie A. Karvonen-Gutierrez, Howard M. Kravitz, Barbara Sternfeld, S. Justin Thomas, Martica H. Hall, Kelley Pettee Gabriel

**Affiliations:** 1 Department of Epidemiology, The University of Alabama at Birmingham, Birmingham, AL USA; 2 Epidemiology Data Center, The University of Pittsburgh, Pittsburgh, PA USA; 3 Department of Epidemiology, The University of Pittsburgh, Pittsburgh, PA USA; 4 Department of Health and Human Development, The University of Pittsburgh, Pittsburgh, PA USA; 5 Division of Research, Kaiser Permanente Northern California, Oakland, CA USA; 6 Center for Behavioral Cardiovascular Health, Columbia University Medical Center, New York, NY USA; 7 Department of Epidemiology, University of Michigan, Ann Arbor, MI USA; 8 Department of Psychiatry and Behavioral Sciences and Department of Preventive Medicine, Rush University Medical Center, Chicago, IL USA; 9 Department of Psychiatry and Behavioral Neurobiology, The University of Alabama at Birmingham, Birmingham, AL USA; 10 Department of Psychiatry, School of Medicine, The University of Pittsburgh, Pittsburgh, PA USA

**Keywords:** Actigraphy, Accelerometer, Sleep, Physical activity, Harmonization, Machine learning, 24-h activity, Time-use epidemiology

## Abstract

**Background:**

Daily 24-h sleep–wake cycles have important implications for health, however researcher preferences in choice and location of wearable devices for behavior measurement can make 24-h cycles difficult to estimate. Further, missing data due to device malfunction, improper initialization, and/or the participant forgetting to wear one or both devices can complicate construction of daily behavioral compositions. The Method for Activity Sleep Harmonization (MASH) is a process that harmonizes data from two different devices using data from women who concurrently wore hip (waking) and wrist (sleep) devices for ≥ 4 days.

**Methods:**

MASH was developed using data from 1285 older community-dwelling women (ages: 60–72 years) who concurrently wore a hip-worn ActiGraph GT3X + accelerometer (waking activity) and a wrist-worn Actiwatch 2 device (sleep) for ≥ 4 days (N = 10,123 days) at the same time. MASH is a two-tiered process using (1) scored sleep data (from Actiwatch) or (2) one-dimensional convolutional neural networks (1D CNN) to create predicted wake intervals, reconcile sleep and activity data disagreement, and create day-level night-day-night pairings. MASH chooses between two different 1D CNN models based on data availability (ActiGraph + Actiwatch or ActiGraph-only). MASH was evaluated using Receiver Operating Characteristic (ROC) and Precision-Recall curves and sleep–wake intervals are compared before (pre-harmonization) and after MASH application.

**Results:**

MASH 1D CNNs had excellent performance (ActiGraph + Actiwatch ROC-AUC = 0.991 and ActiGraph-only ROC-AUC = 0.983). After exclusions (partial wear [n = 1285], missing sleep data proceeding activity data [n = 269], and < 60 min sleep [n = 9]), 8560 days were used to show the utility of MASH. Of the 8560 days, 46.0% had ≥ 1-min disagreement between the devices or used the 1D CNN for sleep estimates. The MASH waking intervals were corrected (median minutes [IQR]: − 27.0 [− 115.0, 8.0]) relative to their pre-harmonization estimates. Most correction (− 18.0 [− 93.0, 2.0] minutes) was due to reducing sedentary behavior. The other waking behaviors were reduced a median (IQR) of − 1.0 (− 4.0, 1.0) minutes.

**Conclusions:**

Implementing MASH to harmonize concurrently worn hip and wrist devices can minimizes data loss and correct for disagreement between devices, ultimately improving accuracy of 24-h compositions necessary for time-use epidemiology.

**Supplementary Information:**

The online version contains supplementary material available at 10.1186/s44167-023-00017-5.

## Background

Time-use movement behaviors (e.g., sleep, sedentary behavior, and physical activity) [1–3] are modifiable factors associated with numerous health outcomes and all-cause mortality [4–8]. Wearable accelerometers are used to measure time-use behaviors in free-living settings for a variety of populations [9, 10]. While there are numerous consumer wearables (e.g., smart watches, fitness monitors) that have the capacity to estimate waking and sleep behaviors, potential issues such as user feedback biases and data extraction limit their research utility [11, 12]. Additionally, consumer device (e.g., Apple, Fitbit, Garmin) accuracy depends on manufacturer and device type and are liable to algorithm changes that may occur numerous times throughout a study and without notification to investigators, creating unmeasurable noise in the data [13–17]. Research-grade devices (e.g., ActiGraph, activPAL, Actiwatch, GENEActiv) provide substantial flexibility over consumer devices for processing and re-processing of data to achieve current best-practices. However, devices for quantifying 24-h time-use behaviors can be placed on different anatomical locations (e.g., hip, wrist, thigh) and can be worn for different amounts of time (e.g., waking only, sleep only, 24 h/day) depending on the primary outcome(s) of interest. For example, researchers only interested in physical activity behaviors may utilize waking hip placements, whereas those interested in sedentary behaviors may want to consider postural positions and therefore need a device with a thigh placement, and those interested in sleep and/or circadian phase may utilize wrist placements [18, 19].

While a single wrist-worn device to capture movement behaviors have become increasingly popular, validity for measuring physical activity across the intensity spectrum against criterion measures (e.g., indirect calorimetry, doubly labeled water) has a wide range of accuracy (r = 0.17–0.93) [20]. This may lead some researchers to implement protocols that have participants switch the device between the hip (during the day) and wrist (at night), potentially increasing participant non-compliance [21], or consider protocols in which participants wear two or more devices concurrently, as in the current study. Other instances when a multiple-device protocol may be necessary include when multiple funded studies are being conducted simultaneously on a single study population, such as independently funded ancillary studies to large prospective cohorts with initial protocols proposing different devices. To reduce participant burden of wearing devices across many weeks of data collection, researchers may need to collaborate on a multiple-device wear protocol occurring over the course of one week, for example.

To characterize 24-h sleep–wake compositions for multiple-device protocols, approaches to harmonizing simultaneously collected data from multiple devices are needed. Unfortunately, data collection in naturalistic settings increases the potential for protocol deviations that undermine accuracy. Specifically, missing data due to device malfunction, improper initialization, and/or the participant forgetting to wear one or both devices on one or more days complicates construction of day-night pairings. Another common issue researchers face is that despite being instructed to remove the device assessing waking activity during sleep periods, it is not uncommon for participants to wear these devices longer than necessary (i.e., to bed), which can inflate estimates of sedentary behavior time. Through this lens, a multiple-device data harmonization process needs to facilitate two types of adjustment when sleep and activity data are joined for 24-h cycle development. First, the frame of reference for a day should be defined as the concatenation of a dynamic sleep–wake interval rather than a constrained period (e.g., midnight to midnight) that may be utilized when behaviors are viewed separately [18, 22]. Second, harmonization should correct any overlap between monitors if there is behavior categorization disagreement, which may address situations where sleep is incorrectly classified as sedentary behavior or non-wear for one device. The latter could result in inaccuracies due to the device (still) recording after it was removed.

Herein, we present the Method for Activity Sleep Harmonization (MASH) process, a novel method that harmonizes data from multiple devices to create coherent sleep-activity pairings. MASH is a multiple device (hip and wrist) harmonization method that addresses many of the issues described above including, missing data (e.g., not wearing one device) or discordant behavior characterization (e.g., one device characterizes sleep whereas the other device characterizes sedentary behavior), while also accommodating both regular and irregular sleep patterns and minimizing data loss. We detail this method using data from 1285 older women who concurrently wore an ActiGraph accelerometer on the hip and an Actiwatch 2 device on the wrist for up to seven days as part of the Study of Women’s Health Across the Nation (SWAN).

## Methods

### Parent study

**Fig. 1:**
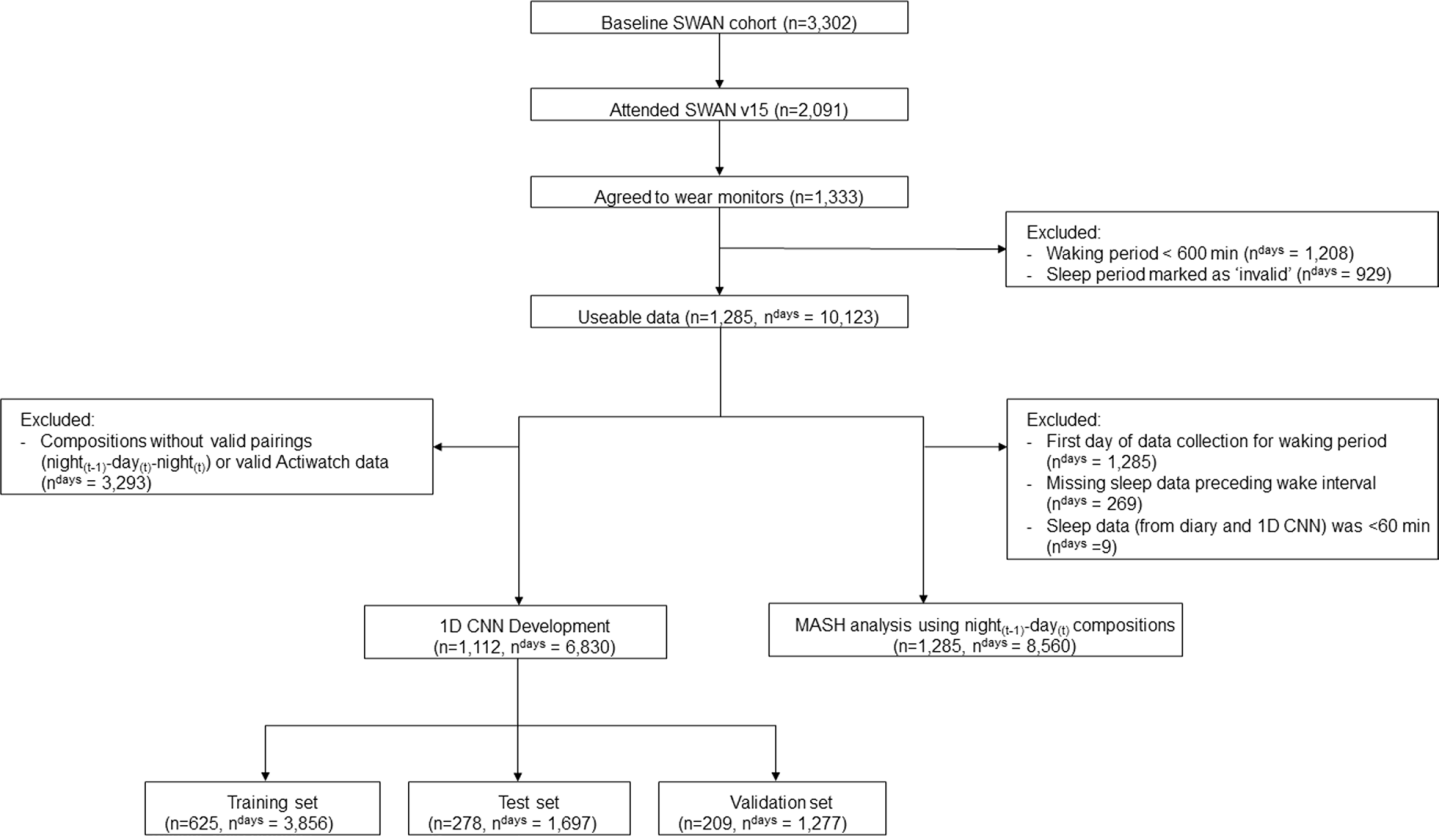
Participant flow diagram for data harmonization

Data are from SWAN, an ongoing longitudinal multisite cohort study of women, which has been previously described [23]. Briefly, 3302 women ages 42–52 years (mean age ± standard deviation [SD]: 46.4 ± 2.7 years) were recruited from seven geographic sites across the U.S.: Boston, MA; Chicago, IL; Southeast area, Michigan; Los Angeles, CA; Newark, NJ; Pittsburgh, PA; and Oakland, CA. Each site recruited White women and women of one other race/ethnicity. Cohort members have been followed through 16 follow-up visits approximately every year. Data for these analyses were collected at the SWAN follow-up visit 15 (2015–2017) (N = 2091 women), in which a subsample of women were invited to concurrently wear two devices to quantify waking and sleep behaviors. A total of 1285 women had valid data for MASH development and evaluation (Fig. 1). Ethics approval was obtained from Institutional Review Boards at each of the seven SWAN sites and all participants provided written informed consent at each visit.

### Data collection

Waking behaviors were quantified using the hip-worn ActiGraph wGT3X+ (*ActiGraph, Pensacola, FL*) device during all waking hours, except for water-based activities, for up to seven days. Raw acceleration data were sampled at 40 Hz and were downloaded and reintegrated to a 60-s epoch using ActiLife6 software [18]. Wear and non-wear (time periods in which participants did not wear the hip device, such as sleeping and water-based activities) were defined using the Choi algorithm with the ‘PhysicalActivity’ R package [24]. Evenson vector magnitude (VM) cut point values [25] were used to classify minutes as sedentary behavior (< 76 VMct·min^−1^), low light (76 to < 903 VMct·min^−1^) intensity (LLPA), high light (903 to < 2075 VMct·min^−1^) intensity (HLPA), and moderate to vigorous intensity physical activity (MVPA) (≥ 2075 VMct·min^−1^). The original 15-s thresholds were multiplied by four to account for the longer epoch (60-s), with slight adjustments to obtain mutually exclusive threshold ranges [26]. For the waking interval, days were classified as adherent if they had ≥ 600 min of wear time. Participants were included if they had ≥ 4 adherent days [18]. These days did not need to be consecutive.

The sleep interval was quantified using the wrist-worn Actiwatch 2 (*Philips Respironics, Murrysville, PA*) device worn for 24 h/day on the non-dominant wrist. Participants completed a diary and were asked to press an event marker on the watch to indicate when they went to bed with the intention to sleep and when they rose from bed for the final time each day. The sleep diary included questions regarding when they got into bed, the time they tried to go to sleep, the time they woke up for the day, and the time when they rose from bed. The Actiwatch was set at 0.05 g for 3–11 Hz and data were sampled in 60-s epochs. To determine total scored sleep time, the Actiwatch 2 data were processed, evaluated for quality, and scored with the event marker, default sleep detection algorithm (wake threshold: 40 ct/min) [27], and the sleep diary (if available) in Actiware 5.0.9 using procedures consistent with the Society of Behavioral Sleep Medicine guidelines [19]. Clock times for sleep onset (beginning of sleep interval) and sleep offset (end of sleep interval) were determined using the start of the first minute (onset) or the last minute (offset) of 10 consecutive minutes of immobility. In addition, all sleep records were visually inspected for quality. Sleep records were removed if there was a Actiwatch malfunction, the Actiwatch was removed prior to sleep (e.g., non-wear), there was no/poor sleep, or there was < 60 min of total scored sleep. These records are henceforth referred to as ‘valid scored sleep data’.

### MASH development

MASH utilizes data from three sources: (1) ActiGraph–count data from Axes 1–3, (2) Actiwatch–lux (white light) and count data, and (3) sleep onset and sleep offset clock times–from the valid scored sleep data corresponding to the beginning (sleep onset) and end (sleep offset) of the primary sleep interval.

Harmonizing the sleep and activity data through MASH is a two-tiered approach that addresses two states of data availability. At its core, MASH reconciles the two datasets (i.e., ActiGraph- and Actiwatch-derived) by determining the bounds of the ‘waking interval’ for each day. The creation of these intervals represents the coherent fusion of the two datasets: night_(t-1)_-day_(t)_-night_(t)_. Any ‘correction’ to the waking behaviors (sedentary behavior, LLPA, HLPA, MVPA) associated with the imposition of these intervals resulted from a disagreement between the sleep and activity data (e.g., the Actiwatch says a person is asleep and the ActiGraph says they are engaging in sedentary behavior).

The first tier of MASH applies to instances where the 24-h period has valid scored sleep data preceding and proceeding it. The wake interval is built using the previous day’s sleep onset time and the current day’s sleep offset time (night_(t-1)_-day_(t)_-night_(t)_). The second tier is used for all other instances where a wake interval does not have valid scored sleep data (e.g., missing sleep onset or sleep offset) immediately surrounding it. This typically occurred for nights that had a Actiwatch malfunction, the Actiwatch was removed, or there was no/poor sleep. In these cases, one-dimensional Convolutional Neural Network (1D CNN) models [28] are used. 1D CNNs were chosen because they have been previously employed on a variety of actigraphy data for algorithm detection [29–31]. The 1D CNN models read the epoch-level ActiGraph and Actiwatch data and assign each epoch with a probability of being ‘within a wake interval’.

Two 1D CNN models were created for MASH. One 1D CNN model accommodates both ActiGraph + Actiwatch data, using data from both devices, and a separate 1D CNN model uses ActiGraph-only data, for situations when the Actiwatch data were invalid or missing. Once the 1D CNN models generate epoch-level predictions, a simple optimization procedure is used to determine which clusters of epochs were most likely to represent the true waking interval. See Additional file 1: Appendix SA for a conceptual framework of the MASH process.

The 1D CNN models were trained using all days that had valid sleep data surrounding them. This sample was randomly divided into training, test, and validation datasets of mutually exclusive individuals. Each 1D CNN model created epoch-level predictions by evaluating centered 101-epoch windows of time surrounding the epoch in question [30]. We considered the costs of misclassifying each epoch as ‘within wake interval’ or ‘outside wake interval’ as equal; therefore, the optimal cutoff probability differentiating these statuses was determined using Youden’s J-statistic [32]. See Additional file 1: Appendix SB and SC for a detailed description of the approaches used to join the sleep and waking datasets and model building.

### Removing the hip device at night prior to sleep onset

While developing MASH, we noticed the predicted sleep intervals resulting from the 1D CNNs were more likely to have shorter waking intervals (both pre-harmonization and after MASH application) and longer sleeping intervals compared to valid scored sleep-derived intervals (e.g., Actiwatch dataset). While it could be that records missing sleep data might have shorter waking intervals because women who were less likely wear the Actiwatch 2 wrist device might not be as diligent at wearing the ActiGraph hip device (thus having shorter wake intervals), having longer sleep intervals is problematic because the act of removing the hip device was being confused with sleep onset.

For records that had valid scored sleep data, the average difference between removing the hip device and sleep onset was 44.4 min. For all records that did not have valid scored sleep data, the average duration of the sleep interval was 45.2 min longer (*P* < 0.001) than the intervals where valid scored sleep data were present. Given the similar sizes of each effect, we therefore used records with valid scored sleep data to construct a bivariate probability distribution for wake interval length by the size of the difference between removing the hip device and sleep onset. The probability distribution was constructed using bounded 2-dimensional kernel density estimation with a minimum value equivalent to what was in the data and an imposed maximum value of 200 min for each variable (~ 3 SD from the mean of 44.4 min). Given the size of each wake interval, the probability distribution was used to generate an estimate of the amount of time that exists between removing the hip device and sleep onset. This estimate was added to the predicted timestamp for sleep onset (thus shortening the duration of the sleep interval).

This process was replicated ten times for all records that did not have scored sleep data indicating sleep onset. The MASH intervals were then constructed using the average of these ten samples. For more information on this process, please consult Additional file 1: Appendix SD and SE. MASH user documentation is available at https://github.com/jsw70/MASH.

### MASH evaluation

The full sample included 10,123 days with useable accelerometry data across 1285 participants. In order to evaluate MASH, for this analysis we focused on days that had full sleep–wake compositions consisting of a sleeping interval followed by a waking interval. This was done to maximize the number of full compositions we could evaluate. For example, while we could have chosen to view a composition as being a day proceeded by night (wake-sleep) this would have led to fewer compositions for evaluation as the last day of data collection would likely be excluded (no sleep data). However, of note, the MASH process creates a bi-directional dataset that allows for flexibility in examining both sleep–wake or wake-sleep compositions.

To evaluate the harmonization process, three exclusions were applied to the sleep–wake data: (1) the first day of data collection for the waking interval was removed because it was a partial day with the first instance of detected wear corresponding to when the devices were distributed and placed on the participant during the in-person exam visit (n = 1285 days), (2) any observation that did not have sleep data preceding the wake interval (n = 269), and (3) any instances where the sleep data was less than 60 min (n = 9). The analytical dataset for the evaluation of MASH included 8560 sleep–wake compositions (Fig. 1).

Potential differences in participant characteristics between the training, test, and validation datasets were assessed using *t*-tests for continuous variables or chi-square tests for categorical variables. Selected participant characteristics included self-reported age (years), race/ethnicity (Black, Chinese, Hispanic, Japanese, White), education (< high school, high school, some college, college, post-college), self-rated health (poor, fair, good, very good, excellent), difficulty walking one mile (yes/no), and obesity (body mass index [BMI] ≥ 30 kg/m^2^) calculated using height and weight at visit 15. Model performance was examined using the C-statistic, i.e., the area under the Receiver Operating Characteristic (AUC-ROC) curve. To account for slight data imbalance (roughly a 66/33 split between ‘within wake interval’ and ‘outside of wake interval’), Precision-Recall curves were also used [33]. Paired *t*-tests were used to examine estimates of time-use movement behaviors between days that were MASH-corrected and when the estimates were not corrected.

## Results

### 1D CNN construction and accuracy

The sample used to build the prediction models included 1112 older women who had both valid scored sleep data preceding and following each wake interval in question. Participants were similarly distributed (*P* > 0.05) for demographics and selected health characteristics across the training (n = 625), test (n = 278), and validation (n = 209) sets (Table 1).

**Table 1:** One-dimensional Convolutional Neural Network (1D CNN) SWAN follow-up visit 15 (2015–2017) participant characteristics, overall and by dataset

Characteristic	Sample (N = 1112)	Training set (n = 625)	Test set (n = 278)	Validation set (n = 209)
	% (n)	% (n)	% (n)	% (n)
Age (M ± SD)	65.5 ± 2	65.4 ± 2	65.2 ± 2	65.8 ± 2
Race/ethnicity				
Black	25.8 (287)	27.8 (174)	23.7 (66)	22.5 (47)
Chinese	12.9 (143)	13.0 (81)	15.1 (42)	9.6 (20)
Hispanic	3.0 (33)	3.0 (19)	2.5 (7)	3.3 (7)
Japanese	12.1 (134)	11.2 (70)	13.3 (37)	12.9 (27)
White	46.3 (515)	45.0 (281)	45.3 (126)	51.7 (108)
Education				
< High school	4.0 (45)	5.0 (31)	2.5 (7)	3.3 (7)
High school	14.9 (166)	13.9 (87)	16.2 (45)	16.3 (34)
Some college	31.3 (348)	28.8 (180)	33.1 (92)	36.4 (76)
College	22.8 (253)	23.5 (147)	21.6 (60)	22.0 (46)
Post-college	26.3 (292)	28.5 (178)	25.2 (70)	21.1 (44)
Missing	0.7 (8)	0.3 (2)	1.4 (4)	1.0 (2)
Obesity (BMI ≥ 30 kg/m^2^)	36.1 (401)	38.2 (239)	32.7 (91)	34.0 (71)
Missing	1.0 (11)	1.0 (6)	1.1 (3)	1.0 (2)
Self-rated health				
Poor, fair, or good	47.3 (526)	47.8 (299)	49.3 (137)	43.1 (90)
Missing	0.8 (9)	1.1 (7)	0.7 (2)	
Difficulty walking one mile	36.5 (406)	39.4 (246)	34.2 (95)	31.1 (65)

*BMI* body mass index

There were no statistically significant differences between the training, test, and validation datasets at the *P* = 0.05 level using *t*-tests for continuous variables or chi-square tests for categorical variables

**Fig. 2:**
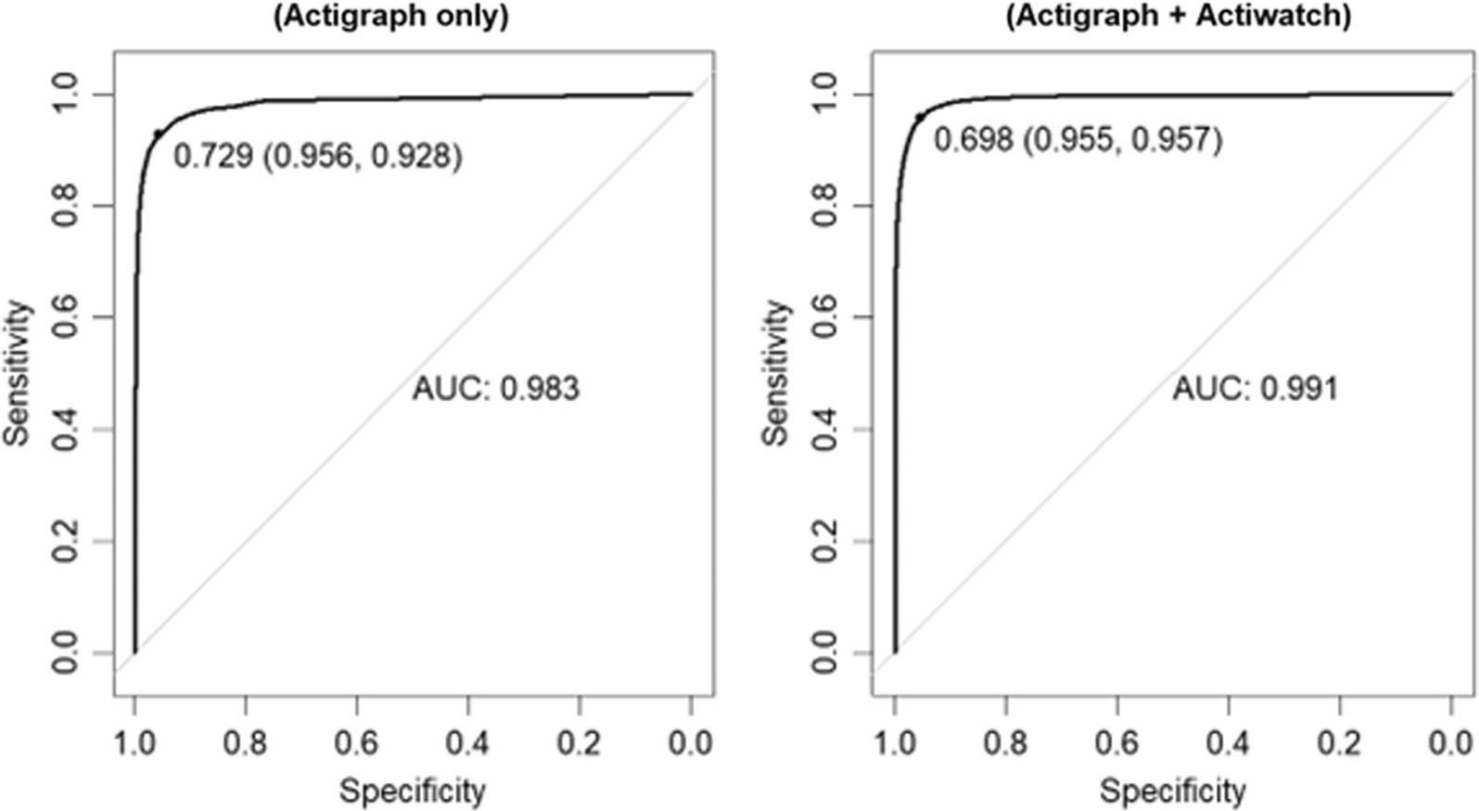
Receiver Operating Characteristic (ROC) curves with cutoff thresholds and sensitivity and specificity values

The AUC-ROC for both 1D CNN models developed for MASH (ActiGraph + Actiwatch or ActiGraph-only) were considered excellent (Fig. 2) with values of 0.991 and 0.983 for the ActiGraph + Actiwatch and ActiGraph-only models, respectively. In addition, the accompanying Precision-Recall AUC were 0.993 and 0.989. Using Youden’s J-statistic to determine a cutoff probability threshold (0.698 and 0.729), the sensitivity of the models at each optimal point was 95.7% for the ActiGraph + Actiwatch model and 92.8% for the ActiGraph-only model. The specificity of the 1D CNN models was 95.5% and 95.6%, respectively.

### Data harmonization

Of the 8560 sleep–wake compositions, 84.9% (n = 7270 records) had valid scored sleep data (i.e., had both sleep onset and sleep offset). Of the remaining 15.1% of days (n = 1290 records), either of the 1D CNN models was applied to estimate (1) sleep offset (n = 32 days), (2) sleep onset (n = 503 days), or (3) both sleep offset and onset (n = 755 days) (Table 2).

**Table 2:** Number of days in the sample, MASH model used, and sleep–wake interval size

Reason	Number of days (N = 8560)	MASH model applied	Sleep–wake interval size, *hours* Mean (SD)
No sleep data are missing	7270	Valid scored sleep	24.04 (1.41)
Sleep data are missing	1290		
Only sleep onset data are missing	503	1D CNN	23.20 (2.35)
Sleep offset data are missing on any day besides first day	32	1D CNN	23.92 (2.52)
Both sleep onset and sleep offset data are missing	755	1D CNN	23.88 (1.68)

*1D CNN* one-dimensional convolutional neural network; *MASH* method for activity sleep harmonization

With the sleep and wake intervals defined, 46.0% (3934 of 8560) of days needed correction to the waking interval. This was to address improper classification of at least one minute-level epoch as both sleep and wake (82.9%; 3262 of 3934 days) or due to missing sleep data (672 of 3934 days).

**Fig. 3:**
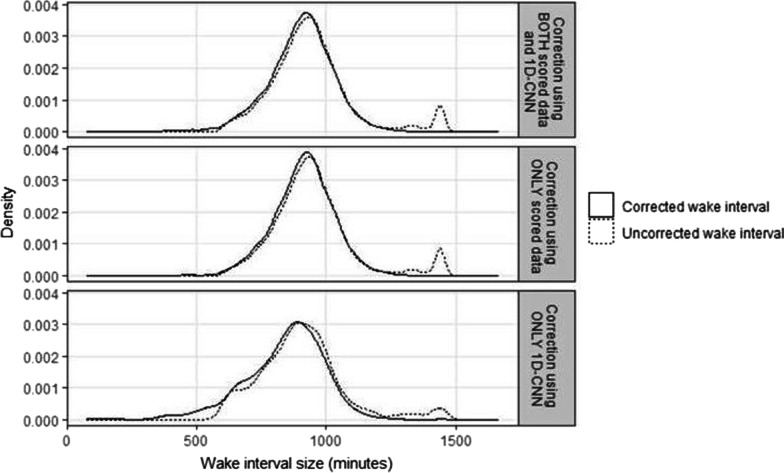
Distribution of wake interval sizes between the uncorrected days and the corrected days, overall and by correction method

For days requiring correction, the average correction applied included a median (interquartile range [IQR]) of − 27.0 (− 115.0, 8.0) minutes of total wake time. The distribution of the MASH-corrected wake intervals smoothed out a cluster of days that the Choi algorithm (applied to ActiGraph data) classified as having > 1200 min (20 h) of waking wear (Fig. 3). This finding is consistent regardless of whether the wake interval was corrected using scored sleep data (median [IQR] = − 21.0 [− 98.0, 12.0] min of total wake time) or 1D CNN (median [IQR] = − 76.0 [− 150.0, − 14.0] min of total wake time) for prediction, even though the distribution of the wake intervals requiring 1D CNN correction were relatively more skewed.

**Fig. 4:**
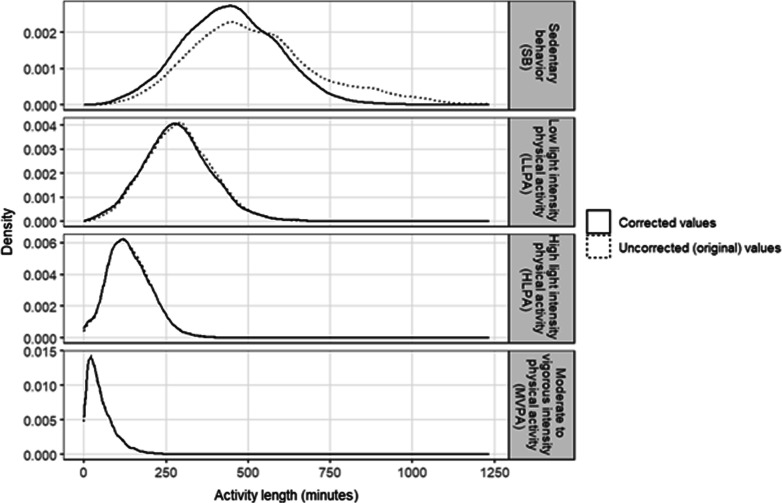
Distribution of waking activity lengths between the uncorrected and corrected activity variables

Table 3 presents the time-use behavior estimates pre-harmonization (e.g., prior to MASH implementation) and the estimates once harmonized using MASH. When compared to other waking behavior estimates (i.e., LLPA, HLPA, and MVPA) the distribution of the sedentary behavior estimate was most influenced once the wear intervals were corrected (Fig. 4). Specifically, sedentary behavior was corrected a median (IQR) of − 18.0 (− 93.0, 2.0) min, whereas the other waking behavior types (LLPA + HLPA + MVPA) were corrected a median (IQR) of − 1.0 (− 4.0, 1.0) min. Paired t-test analysis results demonstrate MASH resulted in statistically significant reductions in all forms of activity; however, only sedentary behavior [*t*(8559) = − 34.2, *P* < 0.001] had a mean difference greater than 3 min. Because the wake interval correction process within MASH also simultaneously creates sleep intervals (in cases where the sleep data are missing), it was not possible to perform t-test analysis on sleep because a substantial portion of the data did not have an ‘uncorrected’ sleep measurement.

**Table 3:** Pre-harmonization and post MASH harmonization time-use estimates (N = 8560) days

Time-use behavior^a^	Pre-harmonization, *minutes* Median (IQR)	MASH harmonization, *minutes* Median (IQR)
Sedentary behavior	438 (344, 547)	419 (329, 512)
Low light intensity physical activity (LLPA)	277 (219, 337)	274 (216, 333)
High light intensity physical activity (HLPA)	137 (100, 181)	136 (99, 181)
Moderate to vigorous intensity physical activity (MVPA)	46 (24, 78)	45 (24, 78)
Sleep^b^	440 (378, 497)	444 (383, 502)

*MASH* method for activity sleep harmonization

^a^Evenson vector magnitude (VM) cut point values were used to classify minutes as sedentary behavior (< 76 VMct·min^−1^), low light (76 to < 903 VMct·min^−1^) intensity (LLPA), high light (903 to < 2075 VMct·min^−1^) intensity (HLPA), and MVPA (≥ 2075 VMct·min^−1^)

^b^Pre-harmonization included 8024 days due to missing data

Participants had a mean (SD) of 6.7 (1.5) sleep–wake compositions. The final MASH harmonized dataset resulted in a mean (SD) sleep–wake composition interval size of 23.97 (1.52) hours. The interval sizes of the sleep–wake compositions were similar across the MASH model applied (Table 2).

## Discussion

Measuring time-use movement behaviors accurately across the continuous 24-h period is critical as these behaviors are interrelated and evidence suggests that the combined effects of these behaviors on health may be greater than their individual effects [34–36]. This had led to 24-h public health guidelines released by the World Health Organization [37] and by some countries (e.g., Australia [38], Canada [39, 40], New Zealand [41], South Africa [42]). Thus, accurate estimation of 24-h sleep–wake cycles, including the contributing behaviors, is paramount. We developed the Method for Activity Sleep Harmonization (MASH) to harmonize time-series data from two accelerometers that use two different placements (wrist and hip) to estimate behaviors comprising the 24-h period. This method creates night-day-night pairings rather than constraining data to a fixed time period (e.g., midnight to midnight). We analyzed an interval of sleep followed by a subsequent waking interval as a 24-h sleep–wake composition. This accounts for the compositional nature of behaviors used in time-use epidemiology [1–3]. Developed on a large sample of older women, the findings suggest the MASH approach (1) minimized data loss due to missing sleep data and (2) improve precision of 24-h sleep–wake compositions. Together, these findings support the utility of MASH to harmonize sleep–wake data obtained from two devices and correct these data, as needed, to more precisely estimate time-use movement behaviors for further analysis.

Physical activity and sleep have largely been separate disciplines, with each preferring certain devices and anatomical placement for field-based data collection. For measuring waking activities, specifically time spent within intensity categories, triaxial accelerometer placement is most accurate at the hip [43, 44]. However, for sleep detection, reliable accelerometry measurement occurs on the wrist [19, 45, 46]. Findings from Full and colleagues suggest estimates of sleep duration using an ActiGraph worn on the hip were significantly higher from polysomnography (PSG), overestimating total sleep time by 37.8 (SD = 61.3) min [47]. This could be the reason the majority of corrections attributed to MASH were to fix instances where sleep was coded as sedentary behavior. Further, total volume of physical activity measured by wrist-worn devices (e.g., Actiwatch 2) have a weak correlation (r = 0.26) with hip-worn devices and thus are not favorable for measuring physical activity [48]. Overestimation of sedentary behavior and underestimation of sleep can have detrimental effects to outcomes research. Sleep, in the absence of disturbances or disorders, is thought to be a restorative and health-promoting process for the body [7], whereas excessive sedentary behavior is associated with several diseases, including sleep disorders (e.g., insomnia, sleep apnea), and excess healthcare costs [8, 49, 50].

Choosing between a single device protocol or a protocol in which participants wear two or more devices concurrently, as in the current study, is largely up to researcher preferences. However, multiple-device protocols may be necessary for independently funded ancillary studies to large prospective cohorts to reduce participant burden of wearing devices across many weeks of data collection. Placing multiple devices at different anatomical locations to increase precision of time-use behaviors has been previously implemented on the hip + thigh [51] and chest + thigh [52]. The MASH method provides an integral step in 24-h time-use assessment by joining accelerometry data from the hip + wrist for increased measurement precision. Prior to this method development, researchers would need to weigh the decision of which behavior outcome could have poor performance accuracy within their study or have participants switch between wear locations, with the possibility of increasing non-compliance [21]. Further, providing open-source code may help with data harmonization and protocol development across studies.

In addition to increasing 24-h measurement precision, MASH also minimizes data loss. MASH evaluates the epoch-level data and applies a classification algorithm that scores the epoch as either within a waking interval or within a sleeping interval. Therefore, although diary/sleep data may be missing, night-day-night pairings could still be constructed. In our sample, a total of 15.1% of days (n = 1290 records) were missing either sleep onset, sleep offset, or both times from the sleep dataset. Without this classification algorithm, those days would be lost during analysis or would need to be imputed. Further, MASH can be used to create daily 24-h compositions rather than a single averaged daily estimate, which can have important implications for examining time-use patterns of behaviors across the week and development of future intervention studies targeting these behaviors.

The limitations of MASH should be noted. This method was developed in a sample of community-dwelling older adult women (age range: 60–72 years). However, we do not believe this would change model development, and given the flexibility and utility of 1D CNN models, implementation of MASH in other studies and populations is achievable. In addition, MASH only removes waking data when the wake interval sizes are longer than the MASH prediction, which occurs in instances where the participant was likely wearing the ActiGraph monitor on the hip while sleeping. MASH is unable to determine activity behavior when data are missing due to not wearing the device during the waking interval, for example, if a participant woke up and did not immediately put on the waking device. We did not calculate these data from the wrist-worn device as the Actiwatch 2 is not accurate for measuring physical activity [48] and we did not impute these minutes as we were unable to determine the true waking activity behavior. However, statistical techniques such as compositional data analysis (CoDA), which treat daily time-use movement behaviors as a composition that are translated to real space through the application of coordinate systems and constrained to 1440 min [53], overcome non-wear issues. With MASH, the period within the resting and sleep intervals (e.g., sleep onset latency) is classified as sedentary behavior, which is supported by the Sedentary Behavior Research Network (SBRN)’s definition as ‘any waking behavior characterized by an energy expenditure ≤ 1.5 metabolic equivalents, while in a sitting, reclining, or *lying* posture’ [54]. However, long sleep latency may have differential effects on health than sedentary behavior [55] and the health-related importance of distinguishing this period requires further study [56]. Lastly, MASH only classifies the main (overnight) sleep interval and did not attempt to classify daytime sleeping (napping) from either device. Despite the limitations, we built MASH and the 1D CNN prediction models using a large sample of women (N = 1285) who wore two devices (hip + wrist) concurrently. The models had excellent classification and there were no significant differences between the scored sleep and 1D CNN prediction models. Using the 1D CNN can help minimize data loss for days when participants forget to wear the wrist device or there was a device malfunction.

## Conclusions

MASH is a dataset harmonization method for merging sleep and waking activity behaviors measured concurrently from multiple devices (hip + wrist). The devices were chosen because of their accuracy in measuring waking activity behaviors (ActiGraph GT3X+) and sleep (Actiwatch 2). We built MASH to merge separate, independent datasets, minimize data loss for missing sleep data, and with the flexibility that this process can be replicated in other studies that simultaneously collect sleep and waking behaviors using two devices. Researchers can use the MASH approach to correct sleep–wake harmonization, construct daily-level compositions, and aggregate to averaged daily values as needed. Ultimately, this approach increases precision of the physical activity and sleep estimates which may improve the accuracy of the observed measures of association with health outcomes.

## Supplementary Information

**Additional file 1:** **SA.** Method of Activity Sleep Harmonization (MASH) conceptual framework. **SB.** Determining valid scored sleep data validity. **SC.** Building the 1D CNN models. **Figure S1.** The network structure for both 1D CNN models with and without the Actiwatch data. **Table S1.** Hyperparameters for both One-dimensional Convolutional Neural Network (1D CNN) models. **Figure S2.** Precision-Recall curves. **SD.** Accounting for the 1D CNN tendency to confuse hip device removal as sleep onset. **Figure S3.** Bivariate probability distribution for the amount of time found between hip device removal and sleep onset for all valid scored sleep data. **Figure S4.** Comparing the distribution of sleep intervals created by the 1D CNN’s and the scored sleep data. The two graphs are separated by whether or not the bivariate sampling was used to adjust for confusing hip device removal with sleep onset. **SE.** Determining wake intervals from epoch-level 1D CNN predictions.

## Data Availability

SWAN provides access to public use datasets that include data from SWAN screening, the baseline visit and follow-up visits (https://agingresearchbiobank.nia.nih.gov/). To preserve participant confidentiality, some, but not all, of the data used for this manuscript are contained in the public use datasets. A link to the public use datasets is also located on the SWAN web site: http://www.swanstudy.org/swan-research/data-access/. Investigators who require assistance accessing the public use dataset may contact the SWAN Coordinating Center at the following email address: swanaccess@edc.pitt.edu.

## Abbreviations

LIPA: Light intensity physical activity
MVPA: Moderate to vigorous intensity physical activity
MASH: Method for Activity Sleep Harmonization
SWAN: Study of Women’s Health Across the Nation
SD: Standard deviation
VM: Vector magnitude
LLPA: Low light intensity physical activity
HLPA: High light intensity physical activity
1D CNN: One-dimensional Convolutional Neural Network
BMI: Body mass index
AUC-ROC: Area under the Receiver Operating Characteristic
IQR: Interquartile range
