# Supplementary material for: Method for Activity Sleep Harmonization (MASH): a novel method for harmonizing data from two wearable devices to estimate 24-h sleep–wake cycles
Source: J Act Sedentary Sleep Behav. 2023 Apr 5;2:8. doi: 10.1186/s44167-023-00017-5 (PMC10492590; doi:10.1186/s44167-023-00017-5)
Supplement: Supplementary file 1 — Additional file 1: SA. Method of Activity Sleep Harmonization (MASH) conceptual framework. SB. Determining valid scored sleep data validity. SC. Building the 1D CNN models. Figure S1. The network structure for both 1D CNN models with and without the Actiwatch data. Table S1. Hyperparameters for both One-dimensional Convolutional Neural Network (1D CNN) models. Figure S2. Precision-Recall curves. SD. Accounting for the 1D CNN tendency to confuse hip device removal as sleep onset. Figure S3. Bivariate probability distribution for the amount of time found between hip device removal and sleep onset for all valid scored sleep data. Figure S4. Comparing the distribution of sleep intervals created by the 1D CNN’s and the scored sleep data. The two graphs are separated by whether or not the bivariate sampling was used to adjust for confusing hip device removal with sleep onset. SE. Determining wake intervals from epoch-level 1D CNN predictions. [file 44167_2023_17_MOESM1_ESM.docx]

Additional files for, ‘Method for Activity Sleep Harmonization (MASH): A novel method for harmonizing data from two wearable devices to estimate 24-h sleep-wake cycles’

1. Method for Activity Sleep Harmonization (MASH) Conceptual Framework


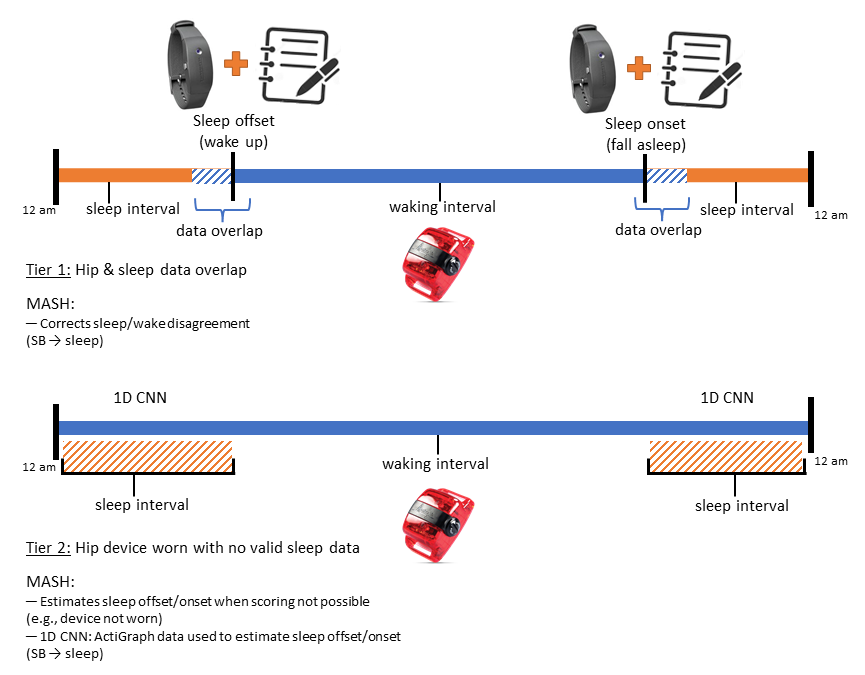


Acronyms: MASH, Method for Activity Sleep Harmonization; SB, sedentary behavior; 1D CNN, One-dimensional Convolutional Neural Network

1. Determining valid scored sleep data validity

Joining the scored sleep data and activity data was particularly problematic because there was no primary key between the datasets and the consecutiveness of days per individual could vary between the two datasets.

Day-night pairings were determined by minimizing the difference between the beginning of the activity day (being midnight of the day) and the time at which sleep interval started. The difference that was minimized had to be less than 1440 minutes (24 hours). To be overly conservative that bad joins were not being confused with irregular/non-traditional sleep schedules, the following conditions would negate a sleep data join and send the data to the One-dimensional Convolutional Neural Networks (1D CNN): (1) A person’s sleep onset was before 8 AM (08:00) for that day (where the wake interval for that day would have to precede this sleep onset time), or (2) a person’s sleep offset was between 4 PM (16:00) in the afternoon and 1 AM (01:00) in the morning.

Out of the 10,123 records available, there were 8,460 records with valid scored sleep data and 1,663 records that were sent to the 1D CNN’s. Of the 1,663 records, only 32 records were the result of meeting the criteria mentioned above.

1. Building the 1D CNN models

The variables used to build the 1D CNN’s are as follows:

1. ActiGraph count data (measuring activity on Axes 1, 2, and 3).
2. Actiwatch count data (one axis labelled ‘Activity’) and light intensity measurements
3. Wearing/non-wearing classifications as determined by the Choi Algorithm.^[[1]](#footnote-1),^^[[2]](#footnote-2)^ Here the Choi algorithm was applied to the ActiGraph data mentioned above.
4. Binary variables indicating if the epoch in question was during the morning (6:00, 13:00), afternoon (13:00, 22:00), or evening (22:00, 6:00). One of the variables was left out to ensure full rank.

For both 1D CNN’s the overall model structure is shown in Figure 1 below. Hyperparameter optimization was performed using the Hyperband function from the KerasTuner API.^[[3]](#footnote-3)^ The hyperband algorithm determines optimal/improved hyperparameters by performing a random search that also simultaneously optimizes resource allocation parameters. The algorithm was set to minimize validation loss (cross-entropy). Due to time constraints, each model underwent ~36 hours of hyperparameter optimization before the process was stopped. Table 1 below shows the resulting parameters for each model, where each layer label is a reference to that found in the network structure in Figure 1.

Figure S1: The network structure for both 1D CNN models with and without the Actiwatch data


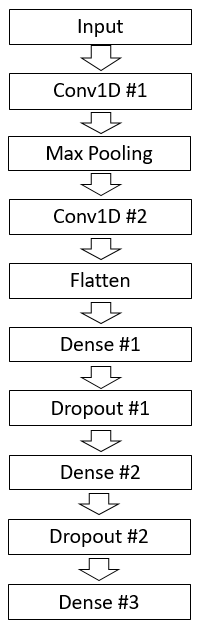


Table S1: Hyperparameters for both One-dimensional Convolutional Neural Network (1D CNN) models

The input for each model was a centered 101-minute window, where the minute being input for prediction had 50 minutes preceding and proceeding it. Due to memory constraints that accompanied such a large dataset, the models were created using a custom data generator. For each batch of the model building process the data generator would read in a single person’s data, sample two random days of their data, create the 101-minute windows for each minute of this subset, and then sample 512 of these centered 101-minute windows.

For each epoch, this process was repeated except the order in which the ID’s were read was randomized each time. When the 512 windows were sampled, there was extra weight put on the minutes within two hours of sleep onset or sleep offset. This was done to encourage the model to focus more on the times surrounding waking up/falling asleep (which, in general are harder to predict than periods of time when a person is in the middle of sleep or being active).

Assessing the 1D CNN accuracy

As was stated in the paper, the 1D CNN models had ROC-AUC values of 0.991 and 0.983 for the models with and without the Actiwatch data, respectively. While the costs of misclassification were equal (thus leading us to use Youden’s J-statistic for determining probability cutoff thresholds), there was a slight imbalance in the original data where people spent roughly 2/3 of their time within a waking interval and 1/3 within a sleeping interval. Given the sampling procedures found within the data generator, it is not clear that this imbalance existed when the model was being built. As a precaution however, we generated Precision-Recall curves to make sure there was no reason to doubt the validity of our ROC-AUC results. Figure 2 below shows the Precision-Recall curves with AUCs of 0.993 and 0.989 for the models with and without the Actiwatch data, respectively.

Figure S2: Precision-Recall curves


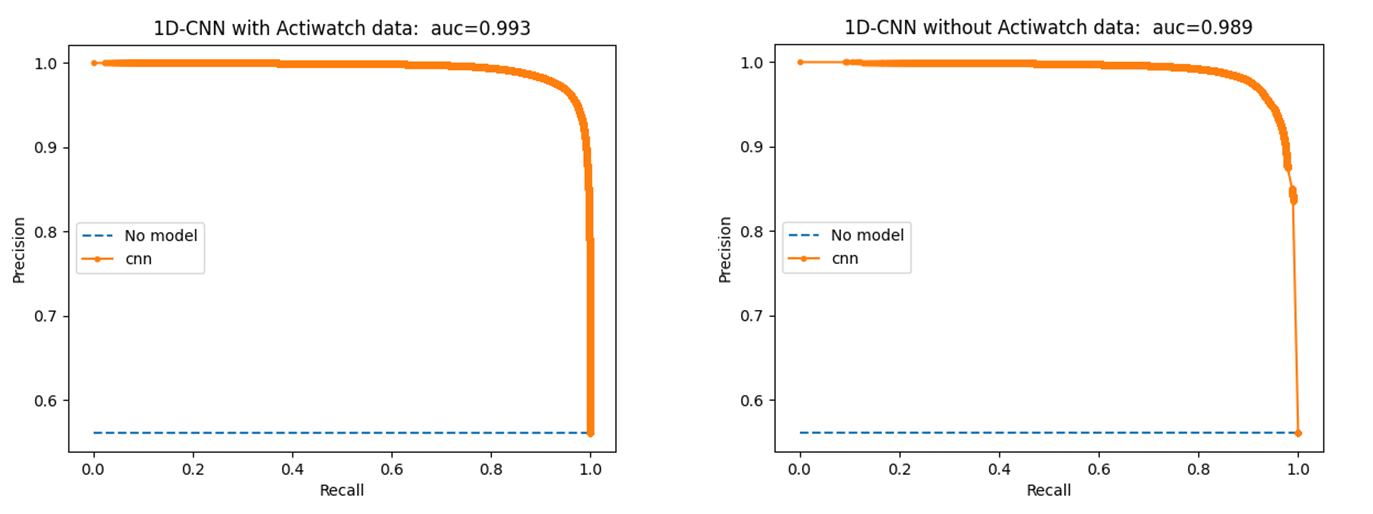


1. Accounting for the 1D CNN tendency to confuse hip device removal as sleep onset

As was described in the paper, there was a substantial amount of evidence to suggest that the 1D CNN models might be confusing hip device removal with sleep onset. To counter this issue, a bivariate probability distribution was used to estimate the potential size of this effect. The bivariate distribution was created from all the data where scored sleep data timestamps regarding the time of sleep onset was available. The two dimensions of this distribution were a) the size of the wearing interval determined by the Choi algorithm, and b) the number of minutes that elapsed removing the hip device and sleep onset. A plot of the distribution can be seen in Figure 3 below.

The difference between hip device removal and sleep onset was sampled 10 times for each observation without scored sleep data for sleep onset and the mean was used in the final analysis of data. Due to the bivariate nature of the distribution, each sample was drawn conditionally on the size of the wearing interval. The logic here was that the size of the effect we were trying to isolate would likely be a function of how much a person wore the hip device during the day.

The distribution of the mean values from these 10 sampling iterations had a median (IQR) = 45 (33, 58) and a mean of 46.5 minutes. The mean of 46.6 minutes is strikingly close to 44.4-minute difference in means between sleep intervals calculated with and without the 1D CNN’s.

Figure 4 below shows the difference in the distribution for sleep interval sizes with and without adjusting for this effect. Using the bivariate probability distribution to adjust for potential instances of confusing hip device removal for sleep onset moves the distribution of sleep intervals leftward, making them much more consistent with the sleep intervals estimated using the scored sleep data.

Figure S3: Bivariate probability distribution for the amount of time found between hip device removal and sleep onset for all valid scored sleep data


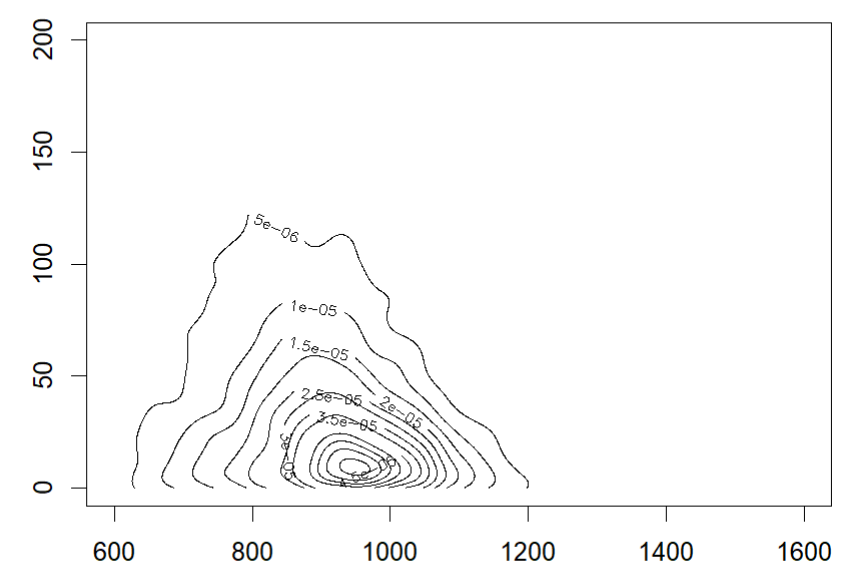


Number of minutes between removing hip device and sleep onset

Number of minutes in wake interval

Figure S4: Comparing the distribution of sleep intervals created by the 1D CNN’s and the scored sleep data. The two graphs are separated by whether or not the bivariate sampling was used to adjust for confusing hip device removal with sleep onset.


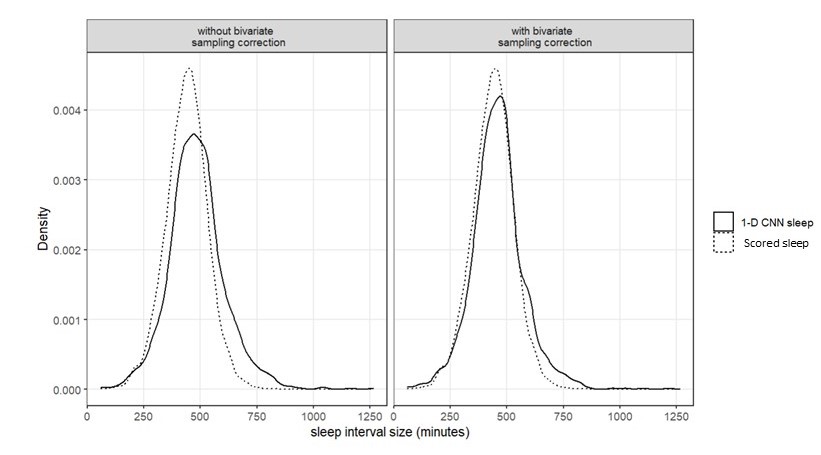


1. Determining wake intervals from epoch-level 1D CNN predictions

Once epoch-level predictions are generated by the 1D CNN models, wake intervals are created by finding the two epochs defining wake-sleep separation that maximizes both the proportion of minutes correctly categorized as being either in the sleep or wake intervals.

The process for determining the wake/sleep intervals goes as follows:

1. Although input for a day is initially restricted to a 24-hour time period, this is extended by 300 epochs at the beginning and end of each day input for prediction. This is done to accommodate the fact that each sleep/wake cycle will not be 24-hours exactly and that many of these cycles could occur at ‘non-traditional’ hours.
2. Each newly defined day is run through their appropriate 1D CNN and epoch-level predictions are generated.
3. The ‘runs’ or periods of being within a waking interval or a sleeping interval are calculated for each day and then a simple algorithm is applied across all possible combinations of these periods to determine which combination maximizes the percentage of correct classifications for being awake and asleep simultaneously. The algorithm is as follows:

$$\min\left( 2-\left( \left( \frac{\# of wake epochs}{total \# of epochs in wake interval} \right)+\left( \frac{\# of sleep epochs}{total \# of epochs in sleep interval} \right) \right) \right)$$

For each person, this process is applied sequentially for each ‘newly defined’ day to ensure that there is no overlap between wake-sleep cycles.

1. Choi L, Beck C, Liu Z, Moore R, Matthews CE, Buchowski MS. PhysicalActivity: Process Accelerometer Data for Physical Activity Measurement. R package version 0.2-4. [↑](#footnote-ref-1)
2. <https://CRAN.R-project.org/package=PhysicalActivity> 2021. [↑](#footnote-ref-2)
3. Li, L., Jamieson, K., Desalvo, G., Rostamizadeh, A., Talwalkar, A. Journal of Machine Learning Research. 18 (2018) pg 1-52. [↑](#footnote-ref-3)
